# Crosslinked Polyethylene (XLPE) Recycling via Foams

**DOI:** 10.3390/polym14132589

**Published:** 2022-06-26

**Authors:** Mohammed Bawareth, Weiheng Xu, Dharneedar Ravichandran, Yuxiang Zhu, Sayli Jambhulkar, Nathan Fonseca, Guillaume Miquelard-Garnier, Visnansky Camille, Lovelady Matthew, William Campbell, Kenan Song

**Affiliations:** 1 Mechanical Systems Engineering, The Polytechnic School (TPS), Ira A. Fulton Schools for Engineering, Arizona State University, Mesa, AZ 85212, USA; mbawaret@asu.edu (M.B.); nfonsec1@asu.edu (N.F.); 2 Systems Engineering, The Polytechnic School (TPS), Ira A. Fulton Schools of Engineering, Arizona State University, Mesa, AZ 85212, USA; weihengx@asu.edu (W.X.); dravich2@asu.edu (D.R.); yzhu226@asu.edu (Y.Z.); sjambhul@asu.edu (S.J.); 3 Laboratoire PIMM, UMR 8006, Arts et Métiers Institute of Technology, CNRS, CNAM, Hesam University, 151 boulevard de L’Hôpital, 75013 Paris, France; guillaume.miquelardgarnier@lecnam.net; 4 Salt River Project (SRP), 1500 N. Mill Ave., Tempe, AZ 85281, USA; camille.visnansky@srpnet.com (V.C.); matthew.lovelady@srpnet.com (L.M.); 5 Rob and Melani Walton Sustainability Solutions Service, Arizona State University, Tempe, AZ 85287-8009, USA; billcampbell@asu.edu; 6 The Polytechnic School (TPS), The School of Engineering of Matter, Transport and Energy (SEMTE), Ira A. Fulton Schools of Engineering, Arizona State University, Mesa, AZ 85212, USA

**Keywords:** crosslinked, polyethylene, polyurethane, recycling, foam, thermal insulator

## Abstract

Efficient recycling of crosslinked polyethylene has been challenging due to manufacturing difficulties caused by chemical crosslinking. This study focuses on simple processing via solid waste powder generation and particle fining for the subsequent crosslinked polyethylene inclusion and dispersion in rigid polyurethane foam. In addition, the concentration effects of crosslinked polyethylene in polyurethane were studied, showing a well-controlled foam microstructure with uniform pores, retained strength, better thermal degradation resistance, and, more importantly, increased thermal capabilities. Thus, the simple mechanical processing of crosslinked polyethylene and chemical urethane foaming showed the massive potential of recycling large amounts of crosslinked polyethylene in foams for broad applications in food packaging, house insulation, and sound reduction.

## 1. Introduction

Cable recycling traditionally focuses on metal recovery (i.e., copper, aluminum), and the plastic cover is usually overlooked. For example, the Salt River Project (SRP) facility (Arizona, USA) annually produces ~540 tons of crosslinked polyethylene (XLPE) plastic waste from electricity wires, and most of this plastic material is currently sent to either landfill or incineration, resulting in land pollution or CO_2_ generation (e.g., every ton of XLPE burnt for heating would generate three tons of CO_2_) [1]. However, commercial XLPE recycling is challenging due to technical and economic reasons. First, a chemical or irradiation process for synthesizing polyethylene (PE) networks achieves high-density crosslinking at the molecular level [2]. Crosslinking substantially improves compression strength, compression set, tensile strength, thermal stability, and water absorption resistance [3]. However, due to covalent bonds forming the network, XLPE behaves as a thermoset that would degrade before melting, posing challenges in mechanical and physical recyclability [4,5,6]. Second, there are currently a few methods of decrosslinking, but their involvement of supercritical fluids or cryogenic gases far exceeds the cost of landfill or incineration [7]. So far, repurposing of XLPE without any chemical modification has only been achieved in a few cases, such as polymer-reinforced concrete pavement [4]. Adding shredded XLPE waste replaces medium-size natural aggregates, enhancing cement mechanical performance [4]. However, the thermal stability of plastics in cement and their potential degradation with the release of toxic gas can raise questions regarding environmental safety and human health. Therefore, there is an urgent need to find an efficient recycling method for turning recycled XLPE into new applications with higher-end values.

Foams are usually a disordered mixture of air cells or bubbles with monodisperse structure or non-uniform pore size and dispersions [8]. The foaming processes have three classifications: mechanical foaming, physical foaming, and chemical foaming [9]. Mechanical foaming occurs by mixing air with polymer resins during or after mechanical stirring. Physical foaming involves thermodynamics regulations due to adjusting temperature and pressure to control polymer microstructures. Chemical foaming is mixing a blowing agent or polymer additive with a matrix to initiate reaction-induced pores. The control over pore size, porosity, and open-/closed-cell morphologies offering reduced weights and superior performance can broaden the foam applications in structural supports, thermal insulators, sound reductions, and biomedical devices. Among all cellular solid materials, the XLPE and rigid polyurethane foam (RPUF) are two primary foaming materials due to their facile processing, low cost, and similar thermal conductivity for insulation purposes [10,11]. The PU foams are obtained by mixing a polyester diol and a diisocyanate, during which procedure the polyol hydroxyl (-OH) and isocyanate functional (-NCO) groups create a urethane linkage reaction [12,13]. These fast-curing and simple reactions without organic solvents are potentially transferrable in XLPE processing as cost-efficient, scalable recycling for thermal barrier or heat dissipation usages [14].

This research explored the possibility of including XLPE in RPUF as a microparticle filler and using them for scalable foams as household insulators. In addition to studies of the mechanical properties, this project mainly explored the thermal behaviors of RPUF/XLPE as a function of compositions and morphologies. This project has leveraged the simple processing of physical separation, pelletizing, and foaming to alter porous microstructures and improve thermal behaviors. As a result, the RPUF/XLPE foams have shown different pore sizes, porosity/density, mechanical behaviors, and thermal properties, with a high potential to serve as thermal insulators.

## 2. Materials and Methods

### 2.1. Materials and Foam Processing

**Figure 1:**
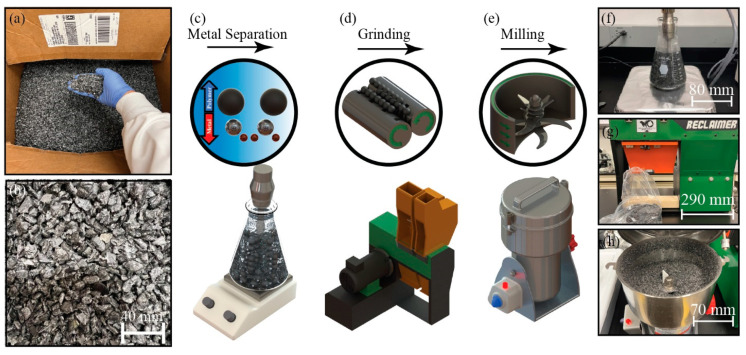
(**a**) XLPE pellets, (**b**) zoom-in with 40 mm scale bar of the XLPE solid waste pellets, plastic waste fining processes for powders and microparticles via (**a**) metal separation from plastics, (**b**) grinding, (**c**) shredding and milling, and (**d**–**h**) the corresponding processing instruments.

XLPE solid waste from insulated stranded cable was provided by Salt River Project (SRP) (Figure 1a,b). These solid wastes in the form of large pellets mainly contain XLPE and metal powders (e.g., copper, aluminum). Subsequently, soaking these mixtures in water via the floating method separated metals from the polymer. In addition, a tip sonicator (SFX250 Sonifier, Branson, Missouri, MO, US) was added to the system to agitate the aluminum particles at 80 °C. The sonication setting was in pulse mode with the on/off frequency of 20/30 s during 2 h and a power amplitude of 175 W (Figure 1c,f). The washed XLPE was stored for 24 h inside a hood with a ventilation system to dry out completely. Then, the dried XLPE was inserted inside a continuous process grinder (Reclaimer, FILABOT, Barre, VT, US) for 1–2 min at 0.75 kW to reduce the particle size (Figure 1d,g) before being inserted into a batch process miller (High-Speed Multi-Function Comminutor, Zhejiang Wanji Plastic Industry Co., Ltd., Zhejiang, China) for 5 min at 2.3 kW, thus forming polymer microparticles (Figure 1e,h).

RPUF was obtained from Smooth-On as the product of FOAM-iT™ 5, which includes two separate components of parts A and B for the foaming reaction. Part A is a composite of s 4,4′ methylene bis(phenyl isocyanate) (MDI) [15], and part B is a composite of ether polyols with silicone surfactant and water [16].

The RPUF/XLPE blend foams were prepared via the following steps. First, the MDI, ether polyols, and XLPE were mixed with mechanical stirring for 45 s. Then, the viscous mixture was poured into different silicon molds with a curing time of 2 h. Consequently, the XLPE microparticles were dispersed during the RPUF foaming reaction. The molds were (*i*) a parallelepiped shape with 30 × 30 mm^2^, (*ii*) a parallelepiped shape with 50 × 50 mm^2^, and (*iii*) a cylindrical shape with 50 mm diameter. After curing, the samples were cut (i.e., a size of (*i*) 8 ± 0.5 mm, (*ii*) 30 ± 0.5 mm, and (*iii*) 40 ± 0.5 mm) with a bandsaw. As a result, the samples were created with XLPE concentrations of 0 wt%, 16.67 wt%, 33.33 wt%, and 47.37 wt%. (*i*) Samples were used to determine the density and thermal properties. (*ii*) Samples were used to assess the thermal response. (*iii*) Samples were used to measure the mechanical properties—other experiments were required to reduce the sample size, such as morphology and thermogravimetric analysis.

### 2.2. Characterization Methods

XLPE Particle Size: Optical images were recorded using a 3D Measurement System (VR-3000 Wide-Area 3D Measurement System, KEYENCE, Japan), with a camera brightness of 200–250 and a magnification of 12×. The images were analyzed by the ImageJ software tool to determine the particle size and size distributions. Then, the data were analyzed statistically to eliminate the outliers by whisker plot before determining the average size and standard deviations.

Foam Density: Twelve samples with four different concentrations were used to measure the foam density. The foam density was obtained from the gravimetric method (Equation (1)), measuring the foam weight via an analytical balance (AB54-S, Mettler Toledo, Columbus, OH, USA) and volume via electronic calipers (Ultra-Cal V, Fowler, Newton, MA, USA). The foam density (*ρ*) was based on a bulk-sized dimension (i.e., *l* is the foam length, *w* is the width, and *t_avg_* is the average thickness) [17].

(1) $$\rho =m_{n}/\left(l\times w\times t_{avg}\right)$$

Foam Morphology: Four samples with four different concentrations were used to capture the foam morphology. The foam morphology was captured from a scanning electron microscope (SEM) (Auriga, Zeiss, Jena, Germany) with the foam surfaces coated with a 15 nm Au/Pd layer to increase conductivity.

Mechanical Properties: Sixteen samples with four different concentrations were used to determine the mechanical properties. The mechanical properties were measured by following the ASTM D1621-16 (i.e., Standard Test Method for Compressive Properties of Rigid Cellular Plastics) standard at a speed rate of 0.0416 mm/s using a universal test machine (5985 Testing Mechanics, Instron, Norwood, MA, USA).

Degradation Analysis: Eight samples with four concentrations were used for thermogravimetric analysis. Degradation starting points were evaluated by a thermogravimetric analyzer (TGA, TA Instruments, New Castle, DE, USA). The tests started from 0 °C to 900 °C with a 10 min/°C heating ramp rate in two environments of air and nitrogen.

Thermal Conductivity: Eight samples with four concentrations were used to quantify the thermal properties. Each sample had four runs. The thermal conductivity (*K*), diffusivity (*α*), and volumetric specific heat (*Q_v_*) were measured by a thermal analyzer (TPS 2500 S, Hot Disk, Gothenburg, Sweden). The disk sensor type was Kapton 5501 with a transient plane source of 4.67 × 10^−3^ K^−1^ and thermal resistance of 6.74 Ω. The probing depth was 6.403 mm, and the measurement time was 40 s. Based on the temperature transient response and residual temperature response, each sample’s heat flow was adjusted to 15–40 mW [18]. Then, the specific heat capacity (*C_P_*) was calculated by Equation (2).

(2) $$C_{p}=Q_{v}/\rho$$

Thermal Responses: The thermal responses were used to calculate the theoretical conductive heat to change temperature. The analysis was based on Fourier’s Law [19,20]. Equation (3) expresses linear conductive heat transfer, *q*(*x*), where *A* is the heated cross-sectional area, t is the sample thickness, and *dT* = *T*_2_ − *T*_1_ is temperature variation between two measurement points. Equation (4) expresses the transient heat transfer as a function of time in any localized point, *q*(*t*), where m is the sample mass and [*T*(*t*) − *T*_0_] is the temperature change at the localized point as a function of time.

(3) q(x)=KA(dT)/t

(4) $$\left[T\left(t\right)-T_{0}\right]=q\left(t\right)/\left(mC_{p}\right)$$

**Figure 2:**
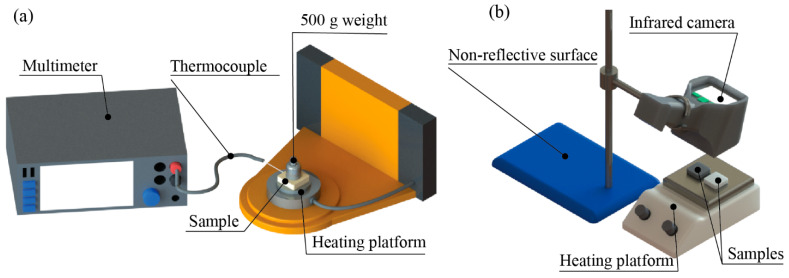
Experiment setups for (**a**) collecting numerical temperature response data and (**b**) thermal images for temperature change.

The thermal responses were measured and recorded by two methods to collect numerical data and thermal images on two samples, RPUF and RPUF/XLPE 33.33 wt%. Each sample had three runs. First, the temperature response data were measured by a digital multimeter (DMM7510, Keithley, Cleveland, OH, USA). Next, the samples were heated by a rheometer (HR-2, TA Instruments, New Castle, DE, USA) (Figure 2a). The test had two configurations: a steady-state environment and fluctuating environment. (*i*) In the steady-state environment, the temperature started at 0 °C with a soaking time of 20 min. Then, the temperature increased to 100 °C at 5 min/°C. Next, the temperature remained constant for 20 min. After that, the temperature decreased to 0 °C by 20 min/°C. The cycle was repeated four times with four different heating increments of 5, 10, 15, and 20 min/°C. (*ii*) In the fluctuating environment configuration, the same procedure of the steady-state configuration was followed but with only one initial soaking time at the first cycle. The thermal images were recorded in heating and cooling by an infrared digital camera (E8, Teledyne FLIR, Wilsonville, OR, USA) (Figure 2b). During heating, all samples were placed simultaneously on a hot plate at 100 °C. During cooling, both samples were placed on a hot plate for 15 min. Then, they were placed on a white non-reflective surface. For every 45 s cycle, thermal images were acquired for heating and cooling experiments.

## 3. Results and Discussion

### 3.1. XLPE Particulate Fining

**Figure 3:**
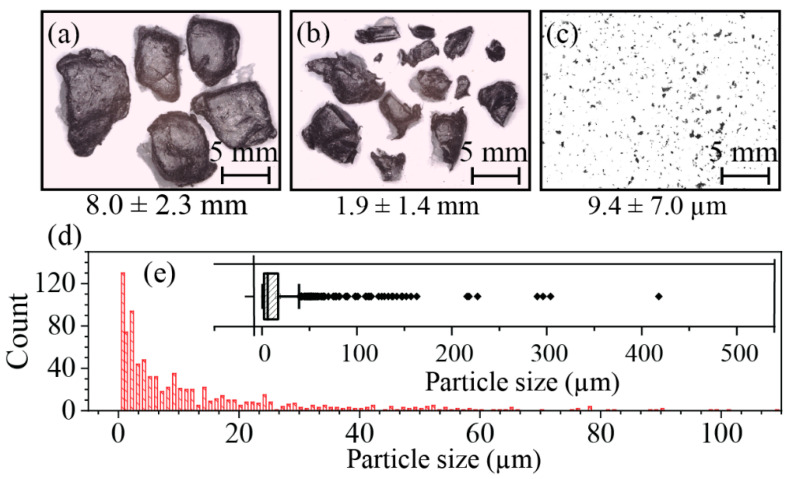
(**a**–**c**) the optical images of fining XLPE to powders for each process and (**d**,**e**) the final particle size distributions.

Foams from extrusion, injection molding, and multilayering usually have the pore sizes distributed at the microscale [21,22,23]. The obtained XLPE wastes have large pellets (i.e., 5–10 mm) that would be hard to include in porous media. Therefore, a series of preprocessing steps were used for fine powder generation. As stated previously, the mechanical treatment used in this study includes metal separation (Figure 1c,f), grinding (Figure 1d,g), and shredding/milling (Figure 1e,h). Optical imaging of samples after these processing steps (Figure 3a–c) and the ImageJ analyses show the efficient XLPE size reduction from 8.0 ± 2.3 mm to 9.4 ± 7.0 µm (Figure 3d,e) [24].

### 3.2. Foam Density

**Figure 4:**
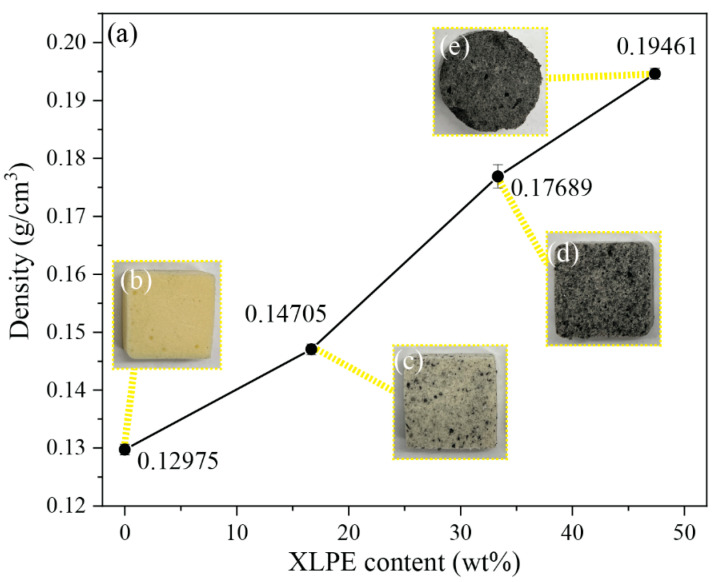
(**a**) RPUF/XLPE density measurements for samples (**b**) RPUF, (**c**) RPUF/XLPE 16.67 wt%, (**d**) RPUF/XLPE 33.33 wt%, and (**e**) RPUF/XLPE 47.37 wt%.

Conventional RPUF and XLPE have a density of 0.05–1 g/cm^3^ and 0.91–0.94 g/cm^3^ [25,26], respectively. The RPUF/XLPE foam density increased with a higher XLPE weight percentage (Figure 4a). The digital photos of these foams also show the contrast between RPUF and XLPE (Figure 4b–e), with varying XLPE concentrations exhibiting distinct pores among samples.

### 3.3. Foam Morphology

**Figure 5:**
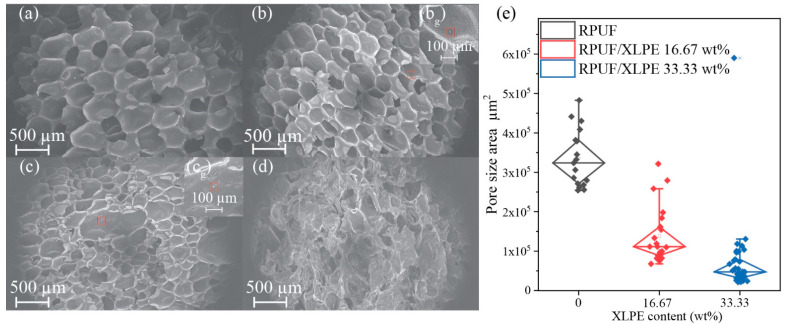
The SEM images for foams of the (**a**) RPUF, (**b**) RPUF/XLPE 16.67 wt%, (**b_g_**) XLPE microparticle fillers with 100 µm scalebar, (**c**) RPUF/XLPE 33.33 wt%, (**c_g_**) XLPE microparticles, and (**d**) RPUF/XLPE 47.37 wt%, (**e**) whisker box plot of pore size area vs. XLPE content.

The foam microstructure influences its mechanical and thermal behaviors, with the SEM images (Figure 5a–d) showing the foam morphology as a function of XLPE addition. Adding XLPE content did not change the foam formability and decreased the pore size (Figure 5e). Additionally, these SEM images show a closed-cell structure consistently, providing unique advantages in insulating heat and enclosing sound waves [27]. Lastly, there is a critical point where the foam morphology loses its pore size uniformity to an undesirable point due to the loss of structural integrity with specific XLPE content (e.g., Figure 5d).

### 3.4. Mechanical Properties

**Figure 6:**
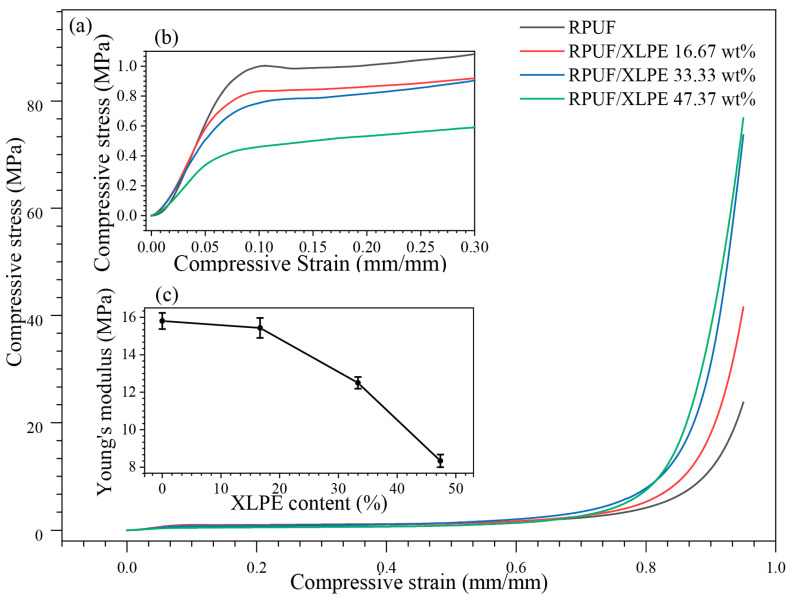
(**a**) Compression tests following the standard ASTM D1621-16 (i.e., Standard Test Method for Compressive Properties of Rigid Cellular Plastics), with the (**b**) zoomed-in elastic regions and (**c**) Young’s modulus as a function of the XLPE content.

The mechanical compression tests in Figure 6 show the XLPE influences on compressive modulus and strength up to a compression strain of 95% (Figure 6a). An increase in XLPE addition decreased the modulus (i.e., 15.8 kPa in RPUF vs. 12.5 kPa in RPUF/XLPE 33.33 wt% XLPE, Figure 6b) but significantly improved the compressive strength under large strains (i.e., 23.8 MPa in RPUF vs. 73.6 MPa in RPUF/XLPE 33.33 wt% XLPE at a strain of 95%) (Figure 6a,b). Experimentally, a higher than 47.37 wt% XLPE in RPUF will disrupt the porous media and degrade the mechanical integrity (e.g., a modulus drop from 15.8 MPa in RPUF foams to 8.3 MPa with a 47.37 wt% XLPE), which is consistent with the morphology (Figure 5d). Furthermore, cell density’s uniformity and circular cell aspect ratio were reconstructed in the foam structure by adding XLPE microparticle fillers to non-uniform cell density and ellipsoidal cell aspect ratio. As a result, the Young’s modulus decreases [28].

### 3.5. Degradation Points

**Figure 7:**
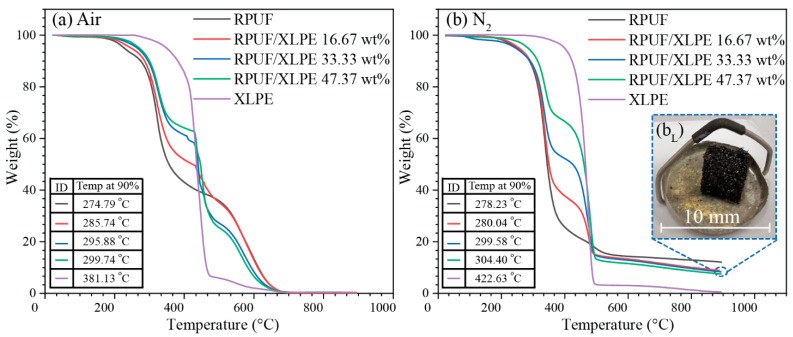
Thermogravimetric (TGA) tests of RPUF with XLPE additions and pure XLPE with the temperature at 10% weight loss in an atmosphere of (**a**) air, (**b**) N_2_, and (**b_L_**) the residue of RPUF/XLPE 47.37 wt%.

TGA tests show the thermal degradation starting points in an atmosphere of air (Figure 7a) and nitrogen (Figure 7b). The transition temperatures at a 10 wt% loss (i.e., Temp at 90%) are listed in Figure 7. XLPE showed higher thermal resistance than RPUF (e.g., ~381 °C for XLPE and ~274 °C for RPUF for the initial thermal degradation in air, Figure 7a); thus, the increase of XLPE in RPUF showed enhanced degradation starting point resistance as compared to RPUF (Figure 7a,b). The air fully degraded the foams to amorphous carbon while the N_2_ retained the foam shape, consistent with the carbon residue ~10 wt% in RPUF/XLPE (Figure 7b_L_). For minimal thermal degradations, the thermal conductivity and response tests were at temperatures < 100 °C.

### 3.6. Thermal Conductivity and Diffusivity Properties

**Figure 8:**
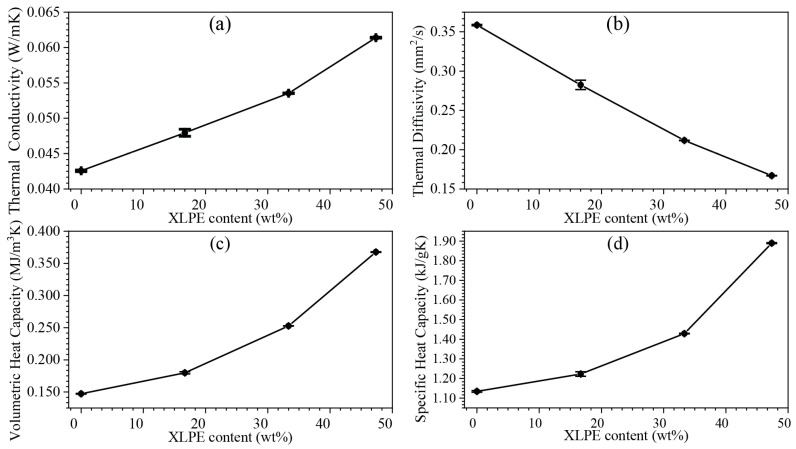
Result of thermal properties of RPUF with varying XLPE percentages: (**a**) thermal conductivity, (**b**) thermal diffusivity, (**c**) volumetric heat capacity, and (**d**) specific heat capacity.

The thermal dissipation of the foams plays an essential role in their applications as thermal barriers. The thermal conductivity increased slightly and quasi-linearly with increasing XLPE ratios (i.e., ~0.04 in RPUF to ~0.06 in RPUF/47.37 wt% XLPE, Figure 8a), showing their potential as thermal insulators that in many applications require *K* under 0.1 W/mK [29]. The thermal conductivity of RPUF/XLPE increases with increasing XLPE content due to the high thermal conductivity of XLPE. Polyethylene’s thermal conductivity could be as high as 20 W m^−1^ K^−1^ [30]. The dispersed XLPE microparticles behaved as energy storage units inside the RPUF. Thermal diffusivity, defined by the thermal conductivity divided by density and specific heat capacity at constant pressure, measures the heat propagation rate from the hot end to the cold end. With increasing XLPE, α decreased, confirming the foam’s capability to prevent quick thermal energy loss (e.g., it is more time-consuming for specific heat to dissipate in RPUF/XLPE than pure RPUF, Figure 8b). The volumetric specific heat and specific heat capacity increased, which shows the amount of heat needed to change the temperature at a specific volume (Figure 8c) and mass (Figure 8d).

### 3.7. Thermal Response

**Figure 9:**
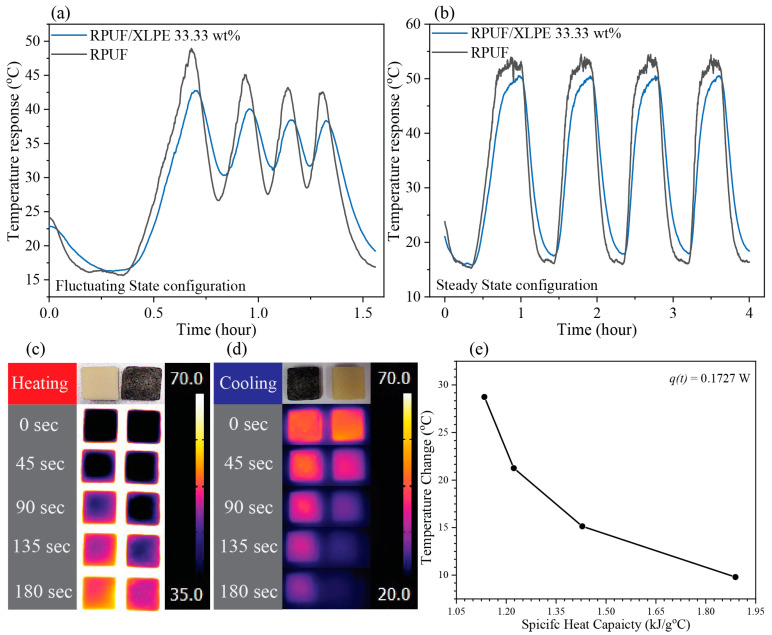
The result of the thermal response between 0 wt% and 33.33 wt% RPUF in XLPE: (**a**) fluctuating-state, (**b**) steady-state; thermal imaging for detecting (**c**) heating, (**d**) cooling history, and (**e**) the theoretical temperature change by conduction.

An in-house setup was built to detect the temperature change as a function of temperature and time in fluctuating-state (Figure 9a) and steady-state (Figure 9b) (Figure 2). In both cases, the RPUF/XLPE 33.33 wt% showed lower temperature changes than the RPUF, consistent with the thermal properties (i.e., *K*, *α*, *Q_v_*, and *C_p_*) in Figure 9. The temperature change in steady-state does not show sufficient improvement in thermal stability relative to fluctuating-state. However, thermal barrier applications such as house insulators and wind turbine nacelle operate in fluctuating-state [31,32,33]. The lower surface temperature within the same heating range (Figure 9c) and the slower cooling rate (Figure 9d) in RPUF/XLPE 33.33 wt% suggested their advantage as a thermal barrier, e.g., in food packaging applications. Concurrently, the theoretical thermal predictions from Equations (3) and (4) indicate that higher thermal conductivity or specific heat capacity leads to lower temperature variations (*dT*) (i.e., with constant thermal energy in the system of 0.173 W, Figure 9e).

## 4. Conclusions

XLPE recycling methods usually require supercritical decrosslinking techniques, which are not yet economically feasible for industrial processing. Our proposed recycling method aimed to optimize the thermal properties, mechanical integrities, manufacturing scalability, and cost-efficiency via simple plastic particle size control and urethane-based foam engineering. The XLPE microparticles were included in RPUF for thermal purposes by reducing the size via mechanical shredding and milling. The initial average XLPE particle size was ~8 mm, reduced to the final XLPE particle size of ~9 µm with enhanced dispersion quality in foams. In addition, the XLPE concentration effects were studied, showing fine morphology control (e.g., pore size and surface roughness), retained mechanical robustness, higher thermal resistance, and better diffusivity parameters. For example, the RPUF/XLPE 33.33 wt% showed a 10% weight loss temperature of ~296 °C, as compared to the 10% weight loss temperature of ~275 °C in RPUF. Similarly, it exhibited a temperature variation of ~12 °C during the fluctuating configuration control, while the RPUF sample was ~22 °C. Thus, this research demonstrated the foaming strategies of RPUF/XLPE compounds as a simple and cost-efficient method of recycling XLPE for thermal applications (e.g., house insulators, automotive headliners, or wind turbine nacelle).

## Data Availability

Data are contained within the article.
